# Emerging Therapeutic Concepts and Latest Diagnostic Advancements Regarding Neuroendocrine Tumors of the Gynecologic Tract

**DOI:** 10.3390/medicina57121338

**Published:** 2021-12-07

**Authors:** Tiberiu-Augustin Georgescu, Roxana Elena Bohiltea, Octavian Munteanu, Florentina Furtunescu, Antonia-Carmen Lisievici, Corina Grigoriu, Florentina Gherghiceanu, Emilia Maria Vlădăreanu, Costin Berceanu, Ionita Ducu, Ana-Maria Iordache

**Affiliations:** 1 Discipline of Pathology, “Carol Davila” University of Medicine and Pharmacy, 020021 Bucharest, Romania; tiberiuaugustin.georgescu@gmail.com (T.-A.G.); lisievici.carmen@yahoo.com (A.-C.L.); 2 Department of Pathology, National Institute for Mother and Child Health “Alessandrescu-Rusescu”, 020395 Bucharest, Romania; 3 Discipline of Obstetrics and Gynecology, “Carol Davila” University of Medicine and Pharmacy Bucharest, 020021 Bucharest, Romania; corigri@gmail.com; 4 Department of Obstetrics and Gynecology, Emergency University Hospital Bucharest, 050098 Bucharest, Romania; octav_munteanu@yahoo.com (O.M.); ionitaducu@gmail.com (I.D.); 5 Discipline of Anatomy, “Carol Davila” University of Medicine and Pharmacy Bucharest, 020021 Bucharest, Romania; 6 Department of Public Health and Management, Faculty of Medicine, “Carol Davila” University of Medicine and Pharmacy Bucharest, 020021 Bucharest, Romania; pbhealth@umf.ro; 7 Department of Marketing and Medical Technology, “Carol Davila” University of Medicine and Pharmacy, 050474 Bucharest, Romania; fgherghiceanu@gmail.com; 8 Faculty of Medicine, “Carol Davila” University of Medicine and Pharmacy Bucharest, 37 Dionisie Lupu, 020021 Bucharest, Romania; vladareanu@gmail.com; 9 Department of Obstetrics and Gynecology, University of Medicine and Pharmacy Craiova, 200349 Craiova, Romania; dr_berceanu@yahoo.com; 10 Optospintronics Department, National Institute for Research and Development in Optoelectronics-INOE 2000, 409 Atomistilor, 077125 Magurele, Romania

**Keywords:** neuroendocrine tumors, gynecologic tract, ovarian carcinoid

## Abstract

Neuroendocrine neoplasms (NENs) are particularly rare in all sites of the gynecological tract and include a variety of neoplasms with variable prognosis, dependent on histologic subtype and site of origin. Following the expert consensus proposal of the International Agency for Research on Cancer (IARC), the approach in the latest World Health Organization (WHO) Classification System of the Female Genital Tumours is to use the same terminology for NENs at all body sites. The main concept of this novel classification framework is to align it to all other body sites and make a clear distinction between well-differentiated neuroendocrine tumors (NETs) and poorly differentiated neuroendocrine carcinomas (NECs). The previous WHO Classification System of the Female Genital Tumours featured more or less the same principle, but used the terms ‘low-grade neuroendocrine tumor’ and ‘high-grade neuroendocrine carcinoma’. Regardless of the terminology used, each of these two main categories include two distinct morphological subtypes: NETs are represented by typical and atypical carcinoid and NEC are represented by small cell neuroendocrine carcinoma (SCNEC) and large cell neuroendocrine carcinoma (LCNEC). High-grade NECs, especially small cell neuroendocrine carcinoma tends to be more frequent in the uterine cervix, followed by the endometrium, while low-grade NETs usually occur in the ovary. NENs of the vulva, vagina and fallopian tube are exceptionally rare, with scattered case reports in the scientific literature.

## 1. Introduction

Neuroendocrine neoplasms (NENs) derive from endocrine cells of the diffuse neuroendocrine system, arising mainly in the gastrointestinal tract, lungs and pancreas [1]. They are rarely encountered in the gynecological tract, and when they are, they involve the ovary or cervix [2]. Historically, the classification schemes for NENs utilized different nomenclature in various organs. Today, pathologists and clinicians rely on the blue books of the IARC-WHO for the classification of NEN in various organs and systems. Rindi et al. attempted the translation of the current system into a more rationale classification framework with specific definitions [3]. NENs are a cancer category and can be classified, based on their biological behavior, in 2 main cancer classes: neuroendocrine tumors (NETs) and neuroendocrine carcinomas (NECs). According to the latest World Health Organization (WHO) Classification System of the Female Genital Tumours, NETs are low-grade or intermediate-grade epithelial neoplasms with morphological and immunohistochemical features of neuroendocrine differentiation. Similar to other body sites, low-grade or grade 1 neuroendocrine tumors are usually referred to as typical carcinoids (TC) and intermediate-grade or grade 2 neuroendocrine tumors are usually referred to as atypical carcinoids (AC). NECs are high-grade (grade 3) and poorly differentiated epithelial neoplasms which show variable morphological and immunohistochemical features of neuroendocrine differentiation. Similar to other body sites, NECs are classified, based on cell morphology, into small cell neuroendocrine carcinomas (SCNEC) and large cell neuroendocrine carcinomas (LCNEC).

This new classification system published in the 5th edition of WHO classification of tumors (WHO) allows both pathologists and clinicians to deal with all NENs in a similar fashion and support further research regarding the prognosis and overall survival of each histological subtype [4].

In this article we review the scientific progress achieved in neuroendocrine neoplasms of the gynecologic tract, since their discovery till today and highlight the latest advancements regarding novel diagnostic modalities, modern histopathologic classifications and most effective therapeutic concepts.

## 2. Materials and Methods

In order to obtain accurate scientific information and achieve comprehensive understanding of these extremely rare entities, we performed multiple search queries on PubMed, Scopus, Embase and clinicaltrials.gov using various combinations between the following terms: “neuroendocrine”, “carcinoid”, “carcinoma”, “small cell”, “large cell” and site-specific keywords, such as: “gynecologic”, “ovary”, “cervix”, “uterus”, “vagina”, “vulva”, “fallopian tube”. As a result, we identified more than 300 unique case reports, short series or literature reviews regarding various forms of neuroendocrine neoplasia of the gynecologic tract, however it may have been defined at that specific point in time, dating as far back as year 1939.

## 3. Results and Discussion

### 3.1. Neuroendocrine Neoplasms of the Cervix

Neuroendocrine neoplasms (NENs) of the cervix were first described in the scientific literature in 1976, by Albores-Saavedra et al., who noticed the histopathological similarities of these tumors with their gastro-entero-pancreatic counterparts [5]. In the cervix, NENs appear to develop from neuroendocrine cells scattered within the glandular and squamous epithelium. Neuroendocrine tumors (NETs) are an exceedingly rare in the cervix, in contrast with the ovary, which is the most frequent site for NETs of the gynecological tract. On the other hand, neuroendocrine carcinomas, particularly small cell neuroendocrine carcinoma (SCNEC) is the most frequent NEN of the cervix.

#### 3.1.1. Well-Differentiated Neuroendocrine Tumors (NETs)

The mean age at diagnosis for cervical carcinoid tumors is approximately 50 years. Carcinoid tumors of the cervix almost never feature the classic paraneoplastic syndromes usually associated with neuroendocrine neoplasms, carcinoid syndrome being extremely rare [6].

In spite of the most recent medical advancements, the absolute scarcity of cervical carcinoids leads to underdiagnosis or misdiagnosis and inappropriate therapeutic management. Ancillary techniques including plasma assays like chromogranin A (CgA) and 5-hydroxyindoleacetic acid (5-HIAA) or immunostaining for neuroendocrine markers may prove to be helpful in some cases, but in order to be requested and actually performed, these methods require increased awareness and a high level of suspicion from both the clinician and the pathologist. Although not correlated with the intensity of flushing, the urinary level of 5-HIAA appears to have increased specificity for carcinoid tumors in comparison with 5-HIAA serum levels [6]. Widespread implementation of Octreoscan significantly enhanced the diagnosis of neuroendocrine tumors, with a median detection rate of approximately 89%, as reported by Modlin et al. [7]. This method uses synthetic radiolabeled somatostatin receptor analogs, which after being injected into the bloodstream of the patient, attach to tumor cells with somatostatin receptors and can be detected via gamma camera scanning.

Microscopically, typical carcinoids (neuroendocrine tumors grade 1) usually feature organoid nested growth pattern, with trabecular, insular or perivascular rosette-like structures. The neoplastic cells have abundant, finely granular, argyrophilic cytoplasm and round to oval nuclei with conspicuous nucleoli, featuring salt and pepper chromatin. There is no or mild cytologic atypia, no necrosis and extremely rare mitoses. Atypical carcinoid tumors (neuroendocrine tumors grade 2) have similar architectural patterns as typical carcinoids, but feature increased cellularity, mild to moderate cytologic atypia, increased mitotic activity (5–10 mitoses/10 high power fields) and foci of necrosis. Lymphovascular space invasion is usually absent.

Carcinoid tumors are usually indolent neoplasms. Most cases of cervical carcinoid reported in the scientific literature were histopathologically diagnosed postoperatively and due to their absolute scarcity, the most effective therapeutic management still remains indefinite. Some authors have reported extremely favorable results in patients diagnosed with typical carcinoid treated solely with radical hysterectomy [8], while others revealed that cisplatin carboplatin and taxol, which are key drugs in gynecological cancer treatment [9], were ineffective for primary atypical carcinoid tumors with metastases [10]. Burzawa et al. achieved complete response using arterial chemoembolization with streptozotocin and 5-fluorouracil in a case of atypical carcinoid of the uterine cervix with multiple liver metastases [11]. In a multi-center retrospective study of neuroendocrine tumors of the uterine cervix, Ishikawa et al. concluded that locally-advanced and extra-pelvic disease are independent prognostic factors [12].

#### 3.1.2. Poorly Differentiated Neuroendocrine Carcinomas (NECs)

Small cell neuroendocrine carcinoma (SCNEC) is a high-grade tumor consisting of small to medium-sized cells with scant cytoplasm and neuroendocrine differentiation. Although rare across the entire gynecological tract, SCNEC is most frequent in the cervix, where it represents approximately 2% of all cervical carcinomas. This tumor has been historically considered a rare subtype of squamous cell carcinoma, but there is now tremendous molecular, immunohistochemical and even serologic evidence for neuroendocrine differentiation.

These tumors affect a wide age range (21–87 years), but tend to be younger than those affected by squamous cell carcinoma. Patients may be asymptomatic, present with vaginal bleeding or abnormal Papanicolaou (PAP) smear. Occasionally, patients may have paraneoplastic manifestations caused by ectopic hormone production, including: Cushing syndrome (corticotropin), syndrome of inappropriate antidiuretic hormone (vasopressin), hypoglycemia (insulin), carcinoid syndrome (serotonin), hypercalcemia (parathormone) or myasthenia gravis. Obviously, the golden standard for diagnosing SCNEC is histopathological examination and immunohistochemical demonstration of neuroendocrine differentiation.

Upon gross inspection, tumors may range from small and inconspicuous to large, ulcerated, sometimes polypoid masses, which may cause marked distortion resulting in a barrel-shaped cervix. Microscopically, SCNEC is typically hypercellular and usually features diffuse, insular, corded or trabecular nested growth pattern with occasional rosette-like or acinar formation. The neoplastic cells are monotonous, small, round or oval to spindle, with scant cytoplasm and hyperchromatic nuclei, featuring finely dispersed chromatin and inconspicuous nuclei. Similar to small cell neuroendocrine carcinoma of the lung, SCNEC may show nuclear molding, crush artefacts, increased mitotic activity (>10 mitoses/10 HPF), numerous apoptotic bodies, extensive areas of necrosis and frequent lymph-vascular space involvement.

Tumor cells show punctate staining for low-molecular weight cytokeratin are also positive for epithelial membrane antigen (EMA), carcinoembryonic antigen (CEA) and neuroendocrine markers. Due to being frequently associated with high-risk HPV infection, particularly HPV 16 and 18, these tumors are generally strongly and diffusely positive for p16. Interestingly, some others have also reported focal TTF-1 positivity in cervical primary SCNEC. Alejo et al. identified HPV in 86% of all cervical NENs, with 55% being positive for HPV 16 and 41% being positive for HPV 18 [13] and a subsequent systematic review and meta-analysis achieved similar results [14]. This particular HPV association is also useful in the differential diagnosis between SCNEC primary to the cervix and metastatic SCNEC, since the latter would not be HPV-positive.

Additionally, Inzani et al. has proved that cervical neuroendocrine carcinomas can express SST2-SST5, which could enable the opportunity for a therapy using somatostatin analogues [15].

The clinical outcome of cervical SCNEC is radically different in comparison to squamous cell carcinoma or adenocarcinoma of the cervix and features more similarities to pulmonary small cell carcinoma. SCNEC is much more likely to feature lymphovascular space invasion, regional lymphatic spread as well as local and distant relapses, with a grim 5-year overall survival rate of approximately 30% [16,17]. Currently available prognostic data suggests that the extent of disease is the most important prognostic factor.

Due to the lack of prospective clinical trials, the therapeutic management of SCNEC is difficult and associated with increased uncertainty. The current National Comprehensive cancer Network (NCCN) Clinical Practice Guidelines in Oncology for SCNEC of the cervix are aligned with the management algorithm proposed by Chen et al. in 2003. Patient workup starts with histopathological examination and imaging data. For tumors ≤4 cm and confined to the cervix, primary treatment consists of radical hysterectomy with pelvic lymphadenectomy and para-aortic lymph node sampling, followed by chemotherapy with cisplatin/etoposide or carboplatin/etoposide in the adjuvant setting. For tumors >4 cm and still confined to the cervix, patients should undergo neoadjuvant chemotherapy first, then consider interval hysterectomy. After radical hysterectomy with bilateral pelvic lymphadenectomy, adjuvant radiotherapy or chemoradiation should be considered. Non-surgical candidates may be referred to chemoradiation and brachytherapy.

Patients with locally advanced disease require chemoradiation, brachytherapy and adjuvant chemotherapy before assessment of treatment response. Tumors with response to treatment undergo surveillance, while persistent or recurrent local disease should receive systemic therapy or consider pelvic exenteration.

Due to the scarcity of these neoplasms and the lack of randomized clinical trials, an individualized treatment approach is appropriate.

Large cell neuroendocrine carcinoma (LCNEC) is a high-grade tumor composed of large cells with neuroendocrine differentiation. LCNEC is rare in the female genital tract but may occur in the cervix and the endometrium. Microscopically, LCNEC is composed of diffuse, insular, organoid, trabecular or cord-like structures, with prominent peripheral palisading and frequent glandular differentiation. The neoplastic cells are medium to large, with abundant eosinophilic/argyrophilic cytoplasm and large vesicular nuclei with prominent nucleoli and brisk mitotic rate (>10 mitoses/10 HPF).

The main differential diagnoses for LCNEC include undifferentiated carcinoma, poorly differentiated squamous cell carcinoma, lymphoepithelioma-like carcinoma, adenocarcinoma with neuroendocrine features, lymphoma and melanoma [18,19]. A broad immunohistochemical panel including p63, CD45 and Melan A, among others, is recommended. Also, before establishing the diagnosis of cervical LCNEC, metastases from other organs should also be taken into consideration.

Most NENs feature positive immunoreaction for at least one neuroendocrine marker: chromogranin A, synaptophysin, neuron specific enolase (NSE), CD56, Leu-7 or PGP9.5. Some SCNECs may be negative for all neuroendocrine markers. Synaptophysin and CD56 are considered the most sensitive markers, although CD56 is nonspecific. If only one neuroendocrine marker is positive, diagnosis of neuroendocrine tumor should be rendered only with a strong suspicion based on morphologic features. Focal immunoreactivity for neuroendocrine markers within a neoplastic epithelial proliferation may be due to the presence of isolated neuroendocrine cells and should not be interpreted as a neuroendocrine neoplastic component. Further studies may ultimately reveal even more specific markers, such as INSM1 (insulinoma-associated protein 1), which has been reported by Kuji et al. as being positive in 95% of NECs [20]. It is important to emphasize that pure neuroendocrine tumors of the cervix are extremely rare, while focal neuroendocrine differentiation may coexist with carcinoma in situ, invasive squamous cell carcinoma or adenocarcinomas. In such cases, p63 can be extremely useful in distinguishing between a NEN and a non-NEN variant of squamous cell carcinoma. If performed, electron microscopy will reveal intracytoplasmatic neurosecretory granules.

LCNEC has an extremely aggressive evolution and most patients do not survive more than 2 years after the initial diagnosis. Various authors propose different therapeutic strategies in the attempt to improve patient outcome. Tangjitgamol et al. studied the expression of estrogen and progesterone receptors in neuroendocrine tumors, but identified that only a minority of patients actually expressed significant amounts of tumor cells positive for hormone receptors [21]. Another novel approach proposed by Kajiwara et al. involved octreotide, a somatostatin type 2A analog, for treating tumors expressing somatostatin type 2A receptors within tumor cells [22].

The therapeutic management of poorly differentiated neoplasms can include, if diagnosed early, a surgical approach, followed by chemotherapy. If the tumoral stage is I or IIA, than a surgical approach can be attempted in patients with tumor size smaller than 4 cm, which can be followed by chemotherapy (etoposide or cisplastin) and radiation therapy [23]. Cases with an early stage, but larger than 4 cm, usually undergo neoadjuvant chemotherapy, followed by surgical excision, once the tumor has shrunken. Patients diagnosed at an advanced stage of disease (IIB-IV), can benefit from combined chemo-therapy and chemoradiation and usually do not undergo a surgical intervention [24]. Both small and large cell neuroendocrine carcinomas of the cervix can benefit from an improved outcome if the therapeutic scheme includes platinum and etoposide [25]. The latter is preferred due to its low toxicity levels [26]. Regarding primary or postoperative chemoradiation, most studies have not been able to show a clear outcome benefit, when comparing to those cases that have not received it [23,27]. Moreover, patients who underwent adjuvant pelvic radiation have also developed subsequent reccurences [28,29].

In a multi-center retrospective study of neuroendocrine tumors of the uterine cervix, Ishikawa et al. discovered that chemotherapy cycle count is associated with patient outcome [12]. Patients with NEC appear to respond well to etoposide & platinum or irinotecan & platinum regimens, while TC shows poor efficacy [12]. According to Hou et al., treatment by radical surgery or definitive radiation therapy with external beam radiation therapy yields equally poor survival [30].

Tempfer et al. suggested that immune checkpoint inhibitors may prove beneficial, but controlled evidence for their efficacy is lacking [31]. In two case reports, nivolumab offered persistent remissions in patients with recurrent disease, as did the MEK-inhibitor trametinib in a woman with recurrent SCNEC and KRAS mutation [32,33].

### 3.2. Neuroendocrine Neoplasms of the Vulva

Vulvar neuroendocrine neoplasms are a heterogenous group of tumors with various histologic findings, different biologic behaviors, very few cases reported in the scientific literature and even less long-term survivors [34]. Based on what has been described so far, vulvar NENs can be subclassified as Merkel cell carcinomas, small cell neuroendocrine carcinomas or large cell neuroendocrine carcinomas. Regardless of their potential differences in etiology and risk factors, neuroendocrine neoplasms of the vulva share similar morphologic appearance and clinical behavior with neuroendocrine neoplasms from other sites of the gynecological tract. In their recent study, Chen et al. reported that high-risk human papilloma virus was positive in all SCNEC and negative in all Merkel cell carcinomas [35]. On the other hand, recent studies regarding Merkel cell carcinoma revealed an extremely strong association with infection by a polyoma virus, termed Merkel cell polyomavirus [36,37].

In the vulva, Merkel cell carcinomas present as a dermal nodule with overlying erythematous surface. Three histopathological variants of Merkel cell carcinoma have been described (trabecular, intermediate and small cell), but there are insufficient case reports in the scientific literature to properly gauge the incidence of each variant in the vulva. Tumors occasionally feature focal squamous or glandular differentiation and may be associated with VIN or invasive squamous cell carcinoma.

Accurate assessment of high-grade neuroendocrine carcinoma is critical for patient management, as all variants are highly aggressive and associated with widespread lymphovascular dissemination, subsequent recurrences and very poor outcome, in general.

Regarding the therapeutical management, Merkel cell carcinomas can be treated through surgical excision, followed by radation therapy. Chemotherapy can also be implemented in patients who suffered reccurences [38]. Small cell carcinoma, as well as large cell carcinoma of the vulva should also be surgically excised, and undergo adjuvant therapy. There are no clear protocols for these cases, but patients can receive either adjuvant chemotherapy with or without radiation therapy and some could even benefit from adjuvant immunotherapy [35].

### 3.3. Neuroendocrine Neoplasms of the Vagina

Primary small cell neuroendocrine carcinoma of the vagina is an extremely rare entity, first reported by Scully et al. in 1984 [39]. Since then, less than 30 cases of small cell neuroendocrine carcinoma have been reported in the scientific literature [40,41]. Due to being extremely rare, there are no specific therapeutic guidelines and most of what is clinically known originates from isolated case reports or is adapted from the therapeutic management of small cell carcinoma of the cervix [25]. Regardless of the histologic variant of vulvar neuroendocrine neoplasia, the absolute scarcity of these tumors warrants definitive exclusion of metastasis from other organs. Kostamo et al. experimented the use of gene expression profiling in order to establish the concentrations of various biomarkers within tumor samples, using quantitative polymerase chain reaction [42]. Using extensive registry data, these methods allow determination of the best likely response to therapeutic options.

Regarding the management of carcinoid tumors of the vagina, surgical excision is the gold-standard, without any additional adjuvant chemotherapy or radiation therapy [25]. To date, there is no clear consensus regarding the therapeutical ap-proach of small cel carcinoma of the vagina. However, usually a multimodal therapy is intended, including a surgical intervention (for small tumors) followed by radiation therapy and chemotherapy. Kostamo et al. has also proposed a possible analysis of the expression of genes that could be targeted through chemoradiation [42].

### 3.4. Neuroendocrine Neoplasms of the Endometrium

Endometrial NETs are exceedingly rare. To date, there are only 3 case reports of primary endometrial NETs in the English scientific literature, all of which were classified as typical carcinoids [43,44]. In one case, the patient presented tumor recurrence approximately eight and a half years after the initial diagnosis and eventually died of intestinal obstruction caused by the carcinoid tumor [44]. We were not able to find any case reports in the scientific literature regarding atypical carcinoid of the endometrium.

Endometrial NECs are more frequent than endometrial NETs, but still infrequent overall, accounting for only 0.8% of all endometrial cancers. Tumors with combined small cell and large cell morphology can be seen and, not uncommonly, they may be associated with other endometrial neoplasms, especially endometrioid carcinoma. This tendency may lead to underdiagnosis of these tumors as poorly differentiated or dedifferentiated carcinomas. The association with other histopathological subtypes of endometrial cancer may indicate that neuroendocrine carcinomas arise from scattered neuroendocrine cells present in type I endometrial cancers or even normal endometrial glands [45]. Another hypothesis is that these tumors could arise from pluripotent stem cells which have intrinsic capacity for both neuroendocrine and endometrioid glandular differentiation. Some authors also described the presence of neuroendocrine differentiation in mixed malignant Müllerian tumors (MMMTs) [46,47]. The distinction of NECs from undifferentiated carcinoma is somewhat arbitrary and relies solely on the percentage of cells expressing at least one neuroendocrine marker.

There are less than 100 cases of endometrial SCNEC reported in the English scientific literature, mainly as case reports and short series of cases [19,34,48]. Studies of LCNEC are even more scarce, with only 36 cases reported in the literature, most of which as tumors with combined small and large cell morphology [48,49,50]. In the largest study to date, the median age at diagnosis was 57 years and patients presented with vaginal bleeding or symptoms related to extra-abdominal metastasis [48]. Paraneoplastic syndromes appear to be uncommon in endometrial NECs. Previous authors reported cases associated with visual paraneoplastic retinopathy [51,52] and Cushing syndrome [53].

The standard treatment for neuroendocrine carcinoma of the endometrium includes total hysterectomy with bilateral salpingo-oophorectomy, followed by combined radiation therapy and chemotherapy (platinum and etoposide) [48]. No adjuvant therapy is necessary for endometrial carcinoid [25].

### 3.5. Neuroendocrine Neoplasms of the Fallopian Tube

To our knowledge, there are only 3 case reports in the English literature regarding NENs of the fallopian tube, all of which classified either as SCNEC or as mixed small and large cell NECs [54,55]. We were not able to identify any NETs primary to the fallopian tube reported in the English literature. NECs of the fallopian tube appear to share similar histopathological characteristics and clinical behavior with other NECs of the gynecologic tract. Due to being so rare, their etiopathogenesis and optimal treatment is yet to be established. Sivridis et al. theorized that SCNEC may arise from mullerian epithelial stem cells [55]. Other potential etiopathogenetic mechanisms include migrational errors of neuroendocrine cells, their implantation during previous surgery, neuroendocrine differentiation of uncommitted stromal cells, or metastasis [54]. Besides a thorough clinical evaluation correlated with patient history and comprehensive imaging studies, there are no other methods or immunomarkers available to date which can reliably distinguish between neuroendocrine neoplasms primary to the fallopian tube and neuroendocrine neoplasms from other sites metastatic to the fallopian tube.

According to Crochet et al., the most reasonable therapeutic attitude might be surgical intervention analogous to that applied in other fallopian tube carcinomas, followed by adjuvant chemotherapy specific for NENs [55]. Poor prognostic indicators include surface involvement, extra-adnexal spread and lymph node metastases.

Neuroendocrine neoplasms of the fallopian tube require total abdominal hysterectomy with bilateral salpingo-oophorectomy and omentectomy, followed by chemo-therapy. The latter usually includes a combination of vincristine, cisplatin and Adriamycin [56]. Other scientific articles report that a cure of six cycles which included carboplatin and etoposide can show a positive evolution of the patient [55]. However, Dursun et al. has reported a case, where the tumor was fully excised but the patient refused any adjuvant therapy, yet the patient survived more than 16 months, without any signs of disease [57]. Standard therapy for carcinoid tumors of the fallopian tube is represented solely by the complete resection of the tumor [58].

### 3.6. Neuroendocrine Neoplasms of the Ovary

Although generally rare within the gynecological system, carcinoid tumor is the most frequent subtype of NEN in the ovary, accounting for 0.1% of all ovarian neoplasms [59,60]. Carcinoid tumor, in general, is one of the predominant subtypes among neuroendocrine tumors, most of them arising in the gastrointestinal and bronchopulmonary systems [61,62,63].

Ovarian carcinoid was first described by Stewart et al. in 1939 [64] and subsequently classified by Kurman et al. as monodermal teratoma in the 4th edition of the WHO [65]. However, the histogenesis of ovarian neuroendocrine tumors still remains unclear. Some authors previously suggested a neural crest origin [66], while others hypothesized midgut derivation for insular and mucinous carcinoid and foregut or hindgut derivation for trabecular and stromal carcinoid [67]. It is now widely recognized that primary ovarian carcinoid frequently occurs on top of ovarian teratoma and less frequently in pure forms. In one of the largest studies on ovarian neuroendocrine tumors to date, Soga et al. reported that approximately 57% of all cases of ovarian carcinoid were associated with teratomaous elements [68]. Other tumors that may be associated with ovarian carcinoid are Brenner tumors and ovarian mucinous tumors [69].

Unlike bronchopulmonary and gastrointestinal carcinoids, which are subdivided into typical and atypical carcinoids based on atypia, necrotic and proliferative activity, ovarian carcinoids are classified according to their cytoarchitectural characteristics into: insular, trabecular, stromal, mucinous and mixed. Regardless of the histologic subtype, ovarian carcinoid features similar cytomorphological characteristics to neuroendocrine tumors from other body sites, including nuclei with minimal atypia and “salt and pepper” chromatin.

The most important diagnostic challenge for the pathologist is distinction between primary ovarian carcinoid and gastrointestinal carcinoid metastatic to the ovary, as both entities show striking overlapping of morphologic features. Primary ovarian carcinoid is usually unilateral and frequently associated with ovarian teratoma, while metastatic carcinoid is often bilateral and does not feature teratomatous elements [70]. Upon gross inspection, metastatic carcinoid has a multinodular appearance, similar to other ovarian metastases, while primary ovarian carcinoid presents as a diffuse homogenous mass. Immunohistochemistry may be useful, but cannot independently distinguish between primary ovarian carcinoid and gastrointestinal carcinoid metastatic to the ovary, as both entities feature extremely similar staining profiles, including positivity for CDX-2 [71]. Other useful diagnostic features which aid in the differential diagnosis of primary vs metastatic, include: patient clinical history, lymphovascular invasion, presence of extraovarian metastatic deposits and post-resection persistence of carcinoid syndrome due to extraovarian tumor. Less challenging differential diagnoses include: Brenner tumor, granulosa cell tumor, Krukenberg tumor or endometrioid carcinoma.

Most ovarian carcinoids have an extraordinarily good prognosis when diagnosed in early stages of disease. Histologic subtype, proliferative activity and pathological stage are the most important prognostic factors. A small subset of tumors, usually represented by insular and mucinous morphology, may have more aggressive behavior and present with extraovarian spread at diagnosis.

Based on current guidelines for ovarian cancer, the therapeutic management for primary ovarian carcinoid should take into consideration several factors, such as: histologic subtype, tumor stage, patient age and desire to preserve fertility [72]. In young patients with incipient disease (stage I), where fertility preservation is preferred, a fertility-sparing surgery with comprehensive staging may be the adequate management. Tumorectomy or unilateral salpingo-oophorectomy with surgical staging appears to be the most reasonable therapeutic option in such cases, as there are multiple evidences of patients with no recurrence after several months of follow-up [73]. Extrapolating prognostic factors valid for epithelial ovarian carcinomas, besides tumor stage, other criteria that should be taken into consideration when considering fertility sparing surgery are maximum tumor diameter, Ki67 proliferative index and presence of lymphovascular invasion. Patients with advanced stages of disease should undergo total hysterectomy with bilateral salpingo-oophorectomy [74]. Chemotherapy may be used in late-stage ovarian carcinoid [75]. Serum serotonin and urinary 5-HIAA may be used to supervise disease progress.

Small cell carcinoma of the ovary is an extremely rare and lethal variant of ovarian carcinoma, which includes two completely different histological subtypes: small cell carcinoma of the ovary, pulmonary type (SCCOPT) and small cell carcinoma of the ovary, hypercalcemic type (SCCOHT) [76]. The fundamental difference between these two subtypes is that SCCOPT is a neuroendocrine neoplasm, while SCCOHT is considered an undifferentiated neoplasm which may feature variable degrees of neuroendocrine immunoreactivity.

Small cell carcinoma of the ovary, hypercalcemic type (SCCOHT) is an extremely rare and lethal tumor which was first described by Scully et al. in 1982. As already mentioned, it is not a neuroendocrine neoplasm per se and we only mention it in this review in regards to its differential diagnostic implications. SCCOHT predominantly affects young women and most cases are associated with symptomatic hypercalcemia. This histological variant of small cell carcinoma of the ovary is associated with SMARCA4 mutations which encode BRG-1 protein, being more closely related to a rhabdoid-like tumor than a NEN [3,77]. Therefore, the two “true” NECs of the ovary are SCCOPT and large cell neuroendocrine carcinoma [4].

There are 23 cases of SCCOPT reported in the scientific literature to date, most of them arising in mature cystic teratomas [78,79,80]. Only 9 out of these 23 cases were reported as pure SCCOPT, others being frequently associated with epithelial tumors like endometrioid carcinoma, mucinous tumor, Brenner tumor. Yin et al. recently reported a primary ovarian small cell carcinoma of pulmonary type associated with endometrial carcinoma in a breast cancer patient receiving tamoxifen [81]. Due to the paucity of data, histogenesis of these tumors remains still unknown.

Tumors may vary in size and are usually solid, with minor cystic components and yellow to tan cut surface. Histopathological examination reveals sheets and nests of small cells with scant cytoplasm, small to medium-sized, oval to elongate nuclei with stippled chromatin and nuclear molding. Trabeculae and rosette-like formation may be seen, as well as extensive single cell apoptosis and necrosis.

The main differential diagnostic challenge of SCCOPT is distinguishing it from SCCOHT as well as pulmonary small cell carcinoma metastatic to the ovary. SCCOPT usually affects perimenopausal women [78], whereas metastases of pulmonary small cell carcinoma occur in younger patients. Hypercalcemia is absent in SCCOPT and tumors are bilateral in almost half of all cases, whereas SCCOHT is bilateral in less than 1% [78] and features characteristic follicle-like spaces.

There is no consensus regarding the appropriate postoperative chemotherapy protocol. In general, adjuvant chemotherapy commonly used to treat pulmonary small cell carcinoma and extrapulmonary uterine and cervical small cell carcinoma may be used. However, there is no supportive evidence that chemotherapeutic agents are effective in treating SCCOPT and most patients usually die of disease within 2 years.

Large cell neuroendocrine carcinoma of the ovary is also an exceedingly uncommon entity, even among NECs. To date, there are less than 60 cases reported in the scientific literature. Based on currently available data, this subtype is extremely aggressive, metastasizes early and may be associated with other epithelial or germ cell tumors [4].

Both small cell and large cell ovarian neuroendocrine carcinomas are frequently associated with non-neuroendocrine carcinoma components and may be defined as mixed neuroendocrine/non-neuroendocrine (MiNENs), similar to those arising in the gastrointestinal or urogenital tract [82].

Collins et al. reported an ovarian mixed tumor, composed out of a large cell neuroendocrine carcinoma and a lesser component of mucinous carcinoma. The authors have postulated that this case is evidence that some neuroendocrine tumors stem from a mucinous tumor (borderline or malignant), without associating a teratomatous component [83]. A similar example has also been reported by Khurana et al., who identified a borderline mucinous ovarian tumor in conjunction to a neuroendocrine carcinoma [84]. In support of this theory comes the article published by Yasuoka et al., who used an X-chromosome clonality assay to demonstrate the monoclonality of both proliferations [85]. Nonetheless, Chenevert et al. has reported a case of a mixed neuroendocrine and non-neuroendocrine carcinoma, that included a mucinous component, but which arose in the background of a teratoma [86].

Regarding the therapeutic options, ovarian carcinoid is usually cured through surgical excision, which can be completed under octreotide administration, in order to prevent a carcinoid crisis. If the patient wishes to preserve her fertility capacity, then a unilateral excision of the ovary can be done. In cases where liver metastases develop, they should also be excised, or at least targeted through cryotherapy/radiofrequency if they are unresectable [87]. There is no need for adjuvant therapy, unless the tumor has recurred or if unresectable, and the patient is also symptomatic. In these cases, the patients can be included in a clinical trial or can receive octreotide therapy [25]. Other chemotherapic agents that have been administered in patients with unresectable carcinoid of the ovary are doxorubicin, cisplatin, etoposide, actinomycin-D, dacarbazine and 5-fluorouracil [25]. Small cell carcinomas of the ovary do not have a clearly established therapy, but most cases are treated through surgical excision followed by chemotherapy (cisplatin and etopiside) [25]. Large cell neuroendocrine carcinomas usually have a similar therapy with six cycles of cisplatin (90 mg/m^2^) and etoposide or paclitaxel (175 mg/m^2^), although some articles have reported recurrences after 10 months [88]. If lymph node metastases are observed, then the patient can also undergo multiple sessions with 75 mg/m^2^ Taxotere every three weeks [88].

### 3.7. Biomarkers in Neuroendocrine Neoplasms of the Gynecological Tract

Regarding the NSE (neuron specific enolase) serum levels in gynecological neuroendocrine tumors, there are little information published in the scientific literature. However, Pang et al. has analyzed the NSE levels prior to the surgical intervention in patients with small cell neuroendocrine carcinoma of the cervix and notices that they can be as high as 370 ng/mL, with variable results between the tested subjects [89]. Chen et al. has observed that four out of six patients with small cell neuroendocrine carcinoma of the cervix have increased NSE serum levels, which ranged up to 154.7 ng/mL, the high levels correlating with the advanced stage of the disease [90]. Other authors observed that the NSE levels decreased in patients with large cell neuroendocrine carcinoma of the uterine corpus from 72 ng/mL to 55 ng/mL after chemotherapy treatment, which included etoposide (100 mg/m^2^), cisplatin (75 mg/m^2^) and octreotide (20 mg IM). In the same case, the biomarker CgA increased its levels from 188 to 249 units/ L after the same therapy [91]. Nonetheless, the impact on the overall survival was poor, the patient succumbing to the disease in less than 12 months. A similar case has been reported by Herraiz et al., who noticed that the NSE levels decreased in a patient with ovarian neuroendocrine carcinoma after neoadjuvant therapy, although the patient developed recurrences one year later [92]. Additionally, of use can be 5-Hydroxyindole acetic acid (5-HIAA), which is known to be a senstivie marker for the identification of a neuroendocrine component in ovarian carcinoma. Although CA-125 is frequently assessed in patients with ovarian carcinoma, it does not show any specificity in cases of neuroendocrine carcinoma [93].

The scientific literature does not provide specific data regarding the serum levels of NSE in gynecological tract carcinoid tumors, but articles that analyzed these levels in carcinoid tumors with other primary location, did not report a clear increase in their values (values ranged between 15–17 ng/mL) [94]. Nonetheless, NSE levels have also been reported as being increased in other gynecological tumors (e.g., ovarian dysgerminoma, immature teratoma) [95,96,97].

### 3.8. Neuroendocrine Differentiation in Non-Neuroendocrine Tumors of the Gynecological Tract

In the cervix, one can encounter MiNEN in which the adenocarcinoma or squamous cell carcinoma component prevails. However, since the neuroendocrine component is the one that will dictate the prognosis of the tumor, this should be the prime element in the pathologic report. If the neuroendocrine differentiation is only focally present, the tumor should still be regarded as a MiNEN, but the non-neuroendocrine component should be the main element in the report [98]. In comparison, in the uterus, scattered neuroendocrine cells can be found in many endometrial carcinomas. Moreover, there are undifferentiated endometrial carcinomas that can express neuroendocrine markers, without having the classic neuroendocrine growth pattern. In order to diagnose a neuroendocrine carcinoma, one should identify the neuroendocrine growth pattern at least in part of the tumor, and neuroendocrine markers should be expressed in more than 10% of the cells [98]. The issue of undifferentiated carcinomas expressing neuroendocrine markers has also been addressed by Rabban et al., who noticed that the presence of neuroendocrine differentiation has no impact on the clinical evolution of these cases [99,100]. When analyzing endometrioid endometrial carcinoma with focal neuroendocrine differentiation, observed only through Grimelius stain or immunohistochemical stains (Chromogranin and synaptophysin), Tamura et al. has observed a worse prognosis for these tumors, when comparing them to those who do not exhibit neuroendocrine differentiation [101]. Regarding the therapeutic impact of neuroendocrine differentiation in endometrial carcinoma, patients should receive the standard therapy, which is usually administered in endometrial carcinoma [100].

## 4. Conclusions

According to the latest edition of the WHO Classification of Tumors, NENs of the gynecological tract are classified, similar to other organs and systems, into low-grade NETs and high-grade NECs. The incidence and clinical outcome of these tumors varies greatly across the gynecological tract. Due to being so uncommon, we also lack sufficient prospective data in order to guide therapeutic decisions.

NETs of the cervix or endometrium are almost never encountered in clinical practice, with only few isolated case reports in the scientific literature. At the other end of the aggressiveness spectrum, SCNEC is the most common neuroendocrine tumor of the cervix.

NECs are highly aggressive tumors, regardless of their site of origin in the gynecological tract. SCNEC is most frequent in the uterine cervix, where it was initially considered an aggressive variant of squamous cell carcinoma.

Appropriate immunohistochemical expression of neuroendocrine markers such as chromogranin, synaptophysin, CD56 and/or NSE is required for a correct diagnosis of NEN. However, due to the relative frequency of the histologic mimickers of NENs, the pathologist must pay close attention to morphologic features of neuroendocrine differentiation and avoid potential pitfalls posed by scattered neuroendocrine cells within adenocarcinomas and squamous cell carcinomas.

We believe that further research regarding the levels of neuroendocrine biomarkers in both NENs and non-neuroendocrine neoplasms with focal neuroendocrine differentiation may provide some insight into the preoperative screening possibilities in patients with tumors of the gynecological tract.

## Data Availability

The datasets used and analyzed during the current study are available from PubMed, ScienceDirect and ISOUG.
